# Comparative evaluation of two DNA methylation assays for triage of hrHPV E6/E7 mRNA–positive women

**DOI:** 10.3389/fpubh.2025.1723553

**Published:** 2025-11-21

**Authors:** Qiuxia Yang, Yongmei Jiang, Fang Liu, Li Chang, Guanglu Che, Shuyu Lai, Jie Teng, Jiaxin Duan, Hui Jian

**Affiliations:** 1 Department of Laboratory Medicine, West China Second University Hospital, Sichuan University, Chengdu, China; 2 Key Laboratory of Birth Defects and Related Diseases of Women and Children (Sichuan University), Ministry of Education, Chengdu, China

**Keywords:** methylation marker, cervical lesion, HPV E6/E7 mRNA, triage, assay

## Abstract

**Background:**

The low specificity of high-risk human papillomavirus (hrHPV) testing necessitates effective triage strategies to avoid unnecessary colposcopy. DNA methylation testing shows promise, but its performance specifically in hrHPV E6/E7 mRNA-positive women, a population with active oncogenic activity, requires further evaluation.

**Methods:**

This study evaluated the clinical performance of two commercial methylation-specific PCR assays, GynTect^®^ and CISCER^®^, as triage tools for detecting high-grade cervical intraepithelial neoplasia (CIN) and cervical cancer in hrHPV E6/E7 mRNA-positive women. A total of 119 women were categorized into five groups based on colposcopy results: cervicitis, CIN1, CIN2, CIN3, and cervical cancer.

**Results:**

Both GynTect^®^ and CISCER^®^ showed increasing detection rates with disease severity, reaching 90.91 and 86.36% in cervical cancer cases, respectively. For CIN2 + lesions, GynTect^®^ demonstrated a detection rate of 59.49%, while CISCER^®^ showed 63.29%, with both assays achieving a specificity of 95.00%. For CIN3 + lesions, GynTect^®^ demonstrated a sensitivity of 73.21%, specificity of 87.30%, a positive predictive value (PPV) of 83.7%, and a negative predictive value (NPV) of 78.6%, whereas CISCER^®^ showed a sensitivity of 76.79%, specificity of 85.71%, a PPV of 82.7%, and an NPV of 80.6%. No statistically significant differences in diagnostic performance were observed between the two assays. Both tests also demonstrated comparable performance across different cytological grades and HPV genotypes, with higher detection rates for HPV 16/18-associated lesions compared to non-16/18 types.

**Conclusion:**

Overall, although limited by its single-center design and modest sample size, GynTect^®^ and CISCER^®^ demonstrated comparable clinical performance for the identification of high-grade CIN in hrHPV E6/E7 mRNA–positive women, supporting their potential role as effective triage tools in HPV-based cervical cancer screening.

## 1 Introduction

Cervical cancer ranks as the fourth most commonly diagnosed cancer and the fourth leading cause of cancer-related mortality among women, with approximately 604,000 newly diagnosed cases and 342,000 deaths worldwide in 2020 (1). Early detection of cervical lesions is valuable, as these lesions develop slowly and typically progress to cervical cancer over a period of more than 10 years (2). Persistent infection with high-risk human papillomaviruses (hrHPVs) has been shown to be causal for the development of cervical precancerous lesions and cancer (3–5). Cytology and hrHPV testing have been widely implemented worldwide for cervical cancer screening due to their high sensitivity.

However, most women infected with HPV will spontaneously clear the infection without developing lesions. The majority of CIN1 and CIN2 regress without treatment. The regression rate of CIN2 lesions is estimated at up to 50%, with even higher regression rates observed in young women aged <30 years (6). Although cytology and hrHPV testing demonstrate high sensitivity for detecting hrHPV infections, their clinical utility is often limited by low specificity, which may result in the detection of regressive CIN2/3 lesions and an increased number of unnecessary colposcopy procedures (7). A highly specific triage strategy, preferably performed on the same sample following an abnormal cytology and/or a positive HPV test, is essential to reduce unnecessary colposcopy referrals (8, 9).

Alterations in DNA methylation, particularly hypermethylation of promoter and 5′ regions of tumor suppressor genes, are early events in HPV-driven carcinogenesis. Persistent hrHPV infection, especially with E6/E7 oncogene expression, interacts with host DNA methyltransferases, resulting in aberrant methylation patterns that silence genes involved in cell cycle regulation and apoptosis. For example, methylation of genes such as PAX1 or ZNF671 can drive lesion progression from low-grade to high-grade CIN and cervical cancer by disrupting epigenetic homeostasis. This epigenetic dysregulation serves as a biomarker of ongoing oncogenic activity and can be detected non-invasively in cervical samples, emerging as a promising approach for triaging hrHPV-positive women and attracting considerable attention in recent years (10, 11). Extensive research has evaluated DNA methylation as a triage method for HPV-positive women, positioning it as a secondary screening strategy alongside conventional approaches, including cytology, HPV16/18 genotyping, and biomarker-based methods such as p16/Ki-67 dual staining (12–15).

Notably, detection of high-risk HPV (hrHPV) E6/E7 mRNA is based on identifying the expression of the viral oncogenes E6 and E7, and it provides a more accurate indication of the oncogenic activity of hrHPV infections than HPV DNA testing, as it reflects active viral gene transcription rather than the mere presence of viral DNA (16). Moreover, the expression of E6 and E7 has been shown to influence host cell epigenetic regulation, particularly by interacting with DNA methyltransferases and altering the methylation profiles of tumor suppressor genes (17). Methylation-based triage tests for hrHPV E6/E7 mRNA-positive women may offer additional improvements in specificity, helping to better distinguish clinically significant lesions from transient infections. However, limited data are available on the performance of methylation assays in this specific population, warranting further evaluation.

Although more than 100 methylation genes have been explored for cervical cancer screening, only a limited number have been developed into commercial assays, with just four methylation-based reagents approved by China’s National Medical Products Administration. Among these, GynTect^®^ (targeting the promoter or 5′ regions of *ASTN1, DLX1, ITGA4, RXFP3, SOX17,* and *ZNF671*) (18) and CISCER^®^ (targeting *PAX1* and *JAM3*) (19) were selected based on their commercial availability, validation in clinical settings, and promising performance in previous studies (20). These assays were chosen to represent multi-gene and dual-gene panels, allowing a comparative evaluation of their triage performance in hrHPV E6/E7 mRNA-positive women. In this study, we conducted a comprehensive comparative analysis of these two assays in 119 hrHPV E6/E7 mRNA-positive cervical samples, aiming to evaluate their effectiveness as triage tools for more targeted and efficient cervical cancer screening and risk stratification.

## 2 Methods

### 2.1 Study subjects

Cervical scrapes were collected from women who visited the colposcopy unit in the Department of Gynaecology at West China Second University Hospital, Sichuan University, between February 2023 and July 2023. The study protocol was approved by the Clinical Research Ethics Committee of West China Second University Hospital, Sichuan University (Medical Research 2024 Ethics Approval (279)). The requirement for informed consent was waived by the Ethics Committee as the study exclusively involved the use of pre-existing, de-identified clinical samples. All patient data are maintained confidentially, and analyses are conducted using de-identified data to ensure privacy protection.

Cytological specimens were independently evaluated by two experienced cytopathologists in accordance with the 2014 Bethesda System. Histopathological slides were independently reviewed by three pathologists in accordance with the WHO Classification of Tumours of Female Reproductive Organs (2014). Cytology and histology evaluations were performed independently and blinded to the methylation results, and methylation testing was conducted without knowledge of cytology or histology outcomes. Cervical samples were collected at the initial visit and initially used for hrHPV E6/E7 mRNA testing on the same day, with the remaining specimens stored at −20 °C. Cytology and histology were typically performed within 1 week of sampling, followed by methylation assays on the stored samples within 2 weeks of collection.

The selection of participants was guided by predefined inclusion and exclusion criteria, ensuring all samples had confirmed cervical biopsy pathology results under colposcopy. The study encompassed 119 women who tested positive for hrHPV E6/E7 mRNA. According to the WHO Classification of Tumours of Female Reproductive Organs (2014), histology represents the gold standard for diagnosing cervical lesions and directly guides clinical management. Although cytology is useful for screening, it is more subjective, whereas histological CIN grading provides an objective assessment of lesion severity and cancer risk. In this study, 119 women were categorized into five groups based on histological findings: 14 with cervicitis, 26 with CIN1, 23 with CIN2, 34 with CIN3, and 22 with cervical cancer (CC). Additionally, 6 hrHPV E6/E7 mRNA-negative specimens were included in the analysis: 2 with cervicitis, 2 with CIN1, 1 with CIN2, and 1 with CIN3. Among the 22 cases of cervical cancer, there were 16 cases of cervical squamous cell carcinoma, 2 cases of cervical adenocarcinoma, 2 cases of cervical carcinoma *in situ*, 1 case of small focal adenoid basal cell carcinoma, and 1 case of cervical small cell neuroendocrine carcinoma. Additionally, the samples were stratified by age (< 30 years and ≥30 years) and by hrHPV infection types (HPV16/18/45 group and non-HPV16/18/45 group).

### 2.2 Inclusion and exclusion criteria

Inclusion criteria: (1) The cervix must be intact, with no history of cervical treatment, including vaginal medication or physical therapy, within the past 3 months; (2) No history of treatment for precancerous lesions or cervical cancer; (3) No history of autoimmune diseases, such as HIV/AIDS.

Exclusion criteria: (1) Pregnant women, those in the postpartum period, or breastfeeding; (2) Individuals who have received radiation therapy or chemotherapy; (3) Known history of or presence of other malignant or borderline tumors in other organs; (4) Use of glucocorticoids, immunosuppressants, or immunomodulatory drugs within the past 6 months.

Sampling criteria: (1) No sexual intercourse 24 to 48 h before specimen collection; no vaginal douching or use of medication within 72 h; (2) Specimen collection should not occur during menstruation.

### 2.3 Aptima HPV assay vs. Aptima HPV 16 18/45 genotype assay

The Aptima HPV mRNA assay (Hologic, San Diego, USA) is designed for semi-quantitative detection of E6 and E7 mRNA expression from 14 high-risk HPV types, including HPV 16, 18, 31, 33, 35, 39, 45, 51, 52, 56, 58, 59, 66, and 68. Subsequently, the Aptima HPV 16 18/45 Genotype Assay is employed to further analyze positive specimens for the presence of HPV 16 and HPV 18/45 genotypes. This assay employs transcription-mediated amplification (TMA) technology to sensitively amplify the target mRNA sequences, followed by detection via a chemiluminescent probe-based hybridization protection assay (HPA). The entire process is fully automated on the Panther System, which integrates RNA extraction, amplification, and detection into a streamlined workflow that minimizes contamination risks. If any high-risk HPV types are detected, the result would be reported as positive. And the system automatically interprets the results, classifying samples as ‘positive (the test value of 0.5 and greater)’ or ‘negative (the test value of < 0.5)’ for high-risk HPV E6/E7 mRNA. Quality controls are embedded throughout the assay to ensure the accuracy and reliability of the test results.

### 2.4 DNA isolation and bisulfite treatment

Cell scrapes were collected in PreservCyt^®^ solution (Hologic, Wiesbaden, Germany). For the GynTect^®^ methylation assay, genomic DNA extraction and bisulfite conversion were performed using the GynTect^®^ reagent and the EpiTect Fast Bisulfite Kit (Qiagen) according to the manufacturer’s instructions. Following elution with 20 μL of elution buffer, 70 μL of water was added, and 80 μL of the diluted DNA was used for each single reaction in the GynTect^®^ real-time PCR assay. In the CisCer^®^ methylation assay, both genomic DNA extraction and bisulfite conversion were carried out using the CisCer^®^ reagent according to the manufacturer’s instructions. After bisulfite conversion, 5 μL of DNA was retained for further analysis. The concentration of genomic DNA was quantified using a Nanodrop 2000 UV–Vis spectrophotometer (PeqLab (VWR Life Science), Erlangen, Germany).

### 2.5 Methylation-specific PCR (GynTect^®^)

The GynTect^®^ assay (oncognostics GmbH, Jena, Germany), which analyses six DNA methylation markers (ASTN1, DLX1, ITGA4, RXFP3, SOX17, ZNF671) and two controls (ACHE, IDS), was performed on all samples according to the manufacturer’s protocol. Each marker was detected using singleplex real-time PCR with EvaGreen (Biotium, USA) as the intercalating dye. For all samples, PCR amplification of individual marker regions was conducted in separate tubes of an eight-well strip, where primers specific to each marker were pre-dried. To perform the real-time PCR, 10 μL of custom-made real-time PCR Master Mix (MM) and 10 μL of the bisulfite-converted DNA was added to each tube.

Real-time PCRs were performed using the ABI7500 PCR system (Life Technologies, Thermo Fischer Scientific, USA). After a 1-min period at 94 °C, 42 cycles at 94 °C for 15 s and 67 °C for 30 s were run, followed by a standard melting curve. Data from the PCR runs were analyzed using ABI Software V2.0.6 and Excel 2019. Samples were considered to be of sufficient quality if the Ct value for the quality control marker ACHE was ≤ 36. For each methylation marker, the Ct value was determined and a delta Ct (Ct_Marker_ – Ct_IDS_) was calculated (prerequisite: Ct value IDS ≤ 42). Methylation markers were scored as positive based on the following criteria: delta Ct ≤ 9 for ASTN1, DLX1, ITGA4, RXFP3, and SOX17, and ≤ 10 for ZNF671. The GynTect^®^ assay was considered positive if the total score of all methylation markers was ≥ 3. The threshold values (ΔCt cut-offs and composite score ≥ 3) are pre-specified according to the manufacturer’s instructions and have been reported in the literature (21). Individual marker scores were assigned as follows: 1 for ASTN1, DLX1, ITGA4, RXFP3, and SOX17, and 3 for ZNF671.

### 2.6 Methylation-specific PCR (CisCer methylation real-time system)

The CisCer^®^ methylation real-time system employs a multiplex methylation-specific real-time PCR to detect the promoter regions of PAX1 and JAM3. The assay was carried out for all samples according to the manufacturer’s instructions (CISPOLY Co., China). In brief, genomic DNA extraction and bisulfite conversion are conducted as detailed in the “DNA isolation and bisulfite treatment” above.

To perform the CisCer^®^ methylation real-time system, 15 μL of custom-made real-time PCR MM, 5 μL of primer mix and 5 μL of template were needed. Real-time PCR reactions were performed under the following conditions: initial denaturation at 96 °C for 10 min; 45 cycles of 15 s at 94 °C, 5 s at 64 °C, and 30s at 60 °C; followed by a final step of 1 min at 25 °C. The assay was considered valid if the Ct value for the quality control marker GAPDH was less than 35. Samples were classified as hypermethylation-positive if the Ct value of PAX1 was ≤ 6.6, or the Ct value of JAM3 was ≤ 10.

### 2.7 Statistical analysis

Statistical analysis was performed using GraphPad Prism (Version 8.00 for Windows, GraphPad Software, San Diego, California, USA). Sensitivity and specificity were estimated to evaluate the diagnostic performance. Exact 95% confidence intervals (CI) were calculated for the proportions assuming a binomial distribution. The chi-square test was employed to statistically compare the performance of the two methylation-based diagnostic assays. A significance level of 0.05 was established for all analyses.

## 3 Results

### 3.1 The general information of different pathological groups

**Figure 1:**
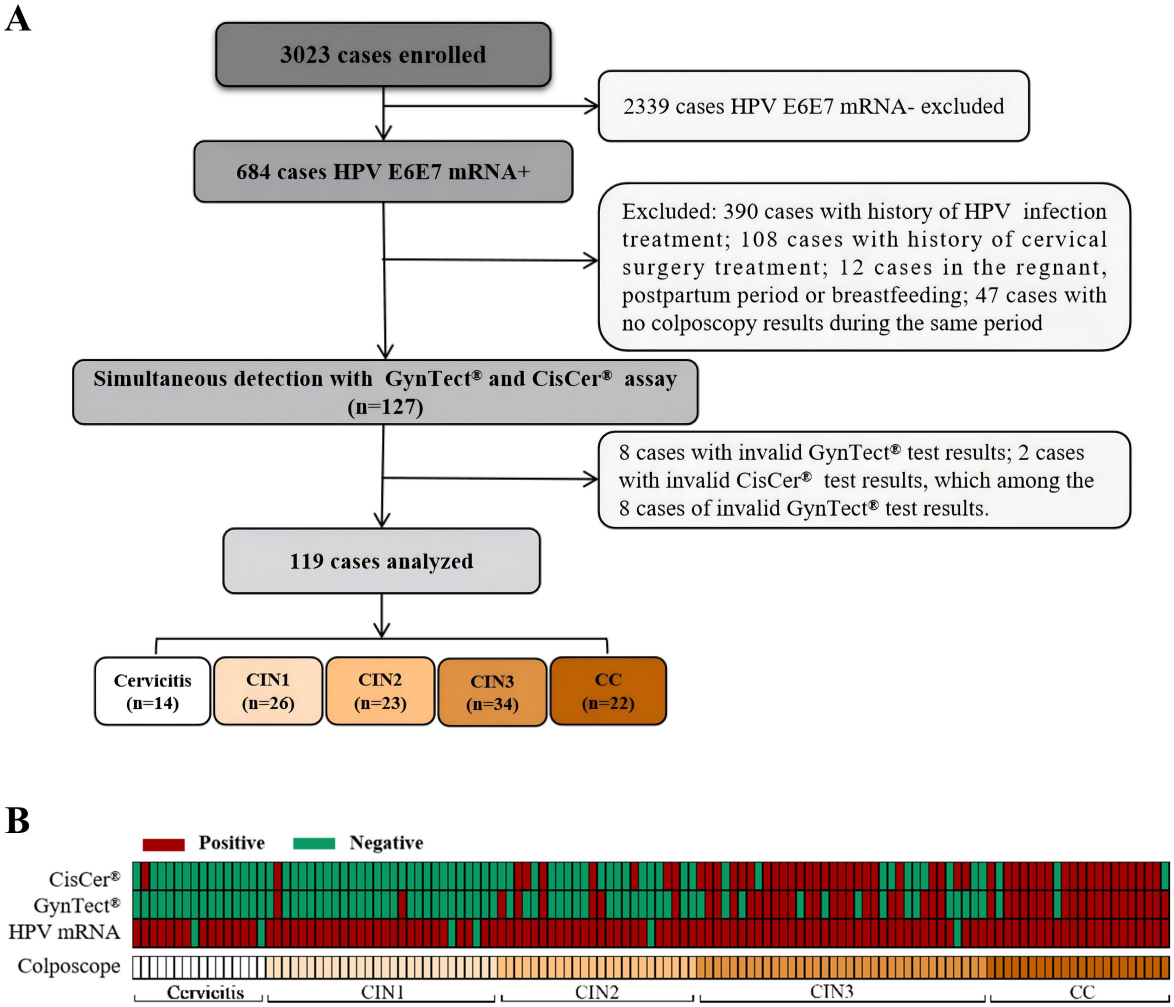
The study’s flowchart and display of relevant results of the specimen. **(A)** The simplified process of the research. **(B)** The results of colposcopy, hrHPV E6/E7 mRNA, GynTect^®^ and CISCER^®^ Methylation corresponding to each specimen. CC, Cervical Cancer; CIN1, Cervical Intraepithelial Neoplasia Grade 1; CIN2, Cervical Intraepithelial Neoplasia Grade 2; CIN3, Cervical Intraepithelial Neoplasia Grade 3.

The study’s flowchart and basic patient information are displayed in Figure 1A and Table 1, respectively. A total of 119 women who tested positive for hrHPV E6/E7 mRNA were included in the study and categorized into five groups based on colposcopy results: 14 with cervicitis, 26 with CIN1, 23 with CIN2, 34 with CIN3, and 22 with cervical cancer (CC), with ages ranging from 21 to 74 years. Notably, the CC group exhibited the highest mean age among the five groups, followed by the CIN3 group. Importantly, we observed that the average hrHPV E6/E7 mRNA RLU/CO values in the CIN1 + groups were significantly higher than those in the cervicitis group. The distribution of HPV genotypes revealed that HPV 16 was most prevalent in the CIN3 and CC groups, while HPV 18/45 was predominant in the CC group. Cytological results varied significantly, with the most severe abnormalities, including HSIL, predominantly observed in the CIN3 and CC groups. In addition, six hrHPV E6/E7 mRNA-negative specimens (two cervicitis, two CIN1, one CIN2, and one CIN3) were also included in the research. Eight samples (missing ΔCt for GynTect^®^ markers) and two samples (GAPDH Ct ≥ 35 for CisCer^®^) failed quality control and were excluded from the analysis. No repeated measurements were performed for these samples.

**Table 1:** The general information of 119 patients with HPV E6/E7 mRNA positive in different pathological groups.

Variable	Cervicitis (*n* = 14)	CIN1 (*n* = 26)	CIN2 (*n* = 23)	CIN3 (*n* = 34)	CC (*n* = 22)	*P*1 value
Age, years						
Mean ± SD	36.07 ± 8.80	39.65 ± 11.95	37.96 ± 11.70	45.35 ± 13.01	53.50 ± 11.55	<0.0001
Range	24–54	22–61	23–59	21–73	30–74	
HPV E6/E7 mRNA						
RLU/CO (Mean ± SD)	9.79 ± 5.53	12.13 ± 5.61	13.50 ± 5.07	13.07 ± 3.90	12.91 ± 4.32	0.0239^a^
HPV 16, *n* (%)	5 (35.70)	6 (23.10)	7 (30.40)	20 (58.80)	12 (54.50)	0.0008
HPV 18/45, *n* (%)	2 (14.30)	1 (3.80)	1 (4.30)	1 (2.90)	6 (27.30)	
Other HPV, *n* (%)	7 (50.00)	19 (73.10)	15 (65.20)	13 (38.20)	4 (18.20)	
Cytology						
NILM, *n* (%)	5 (35.70)	7 (26.90)	0 (0.00)	2 (5.90)	3 (13.60)	0.0003
ASC-US, *n* (%)	6 (42.90)	13 (50.00)	7 (30.40)	7 (20.60)	2 (9.10)	
LSIL, *n* (%)	3 (21.40)	6 (23.10)	6 (26.10)	6 (17.60)	2 (9.10)	
ASC-H, *n* (%)	0 (0.00)	0 (0.00)	2 (8.70)	0 (0.00)	1 (4.50)	
HSIL, *n* (%)	0 (0.00)	0 (0.00)	2 (8.70)	14 (41.20)	3 (13.60)	
Unknown, *n* (%)	0 (0.00)	0 (0.00)	6 (26.10)	5 (14.70)	11 (50.00)	
GynTect^®^						
Positive, *n* (%)	0 (0)	2 (7.69)	6 (26.09)	21 (61.76)	20 (90.91)	<0.0001
Negative, *n* (%)	14 (100.00)	24 (92.31)	17 (73.91)	13 (38.24)	2 (9.09)	
CISCER^®^						
Positive, *n* (%)	1 (7.14)	1 (3.85)	7 (30.43)	24 (70.59)	19 (86.36)	<0.0001
Negative, n (%)	13 (92.86)	25 (96.15)	16 (69.57)	10 (29.41)	3 (13.64)	
*P*2 value	1	1	1	0.6088	1	

The P1-values were obtained by comparing different pathological groups using ANOVA or chi-square tests. The P2-values were obtained by comparing GynTect^®^ with CISCER^®^ in every pathological group using Fisher’s exact test.

a Means HPV E6/E7 mRNA RLU/CO in cervicitis compared with CIN1 + .

### 3.2 Methylation positive rate in two assays based on colposcopy results

Methylation positive rates for GynTect^®^ and CISCER^®^ varied across different pathological groups (Figure 1B and Table 1). Specifically, the methylation-positive rate of GynTect^®^ increased from 0% in the cervicitis group to 90.91% in the CC group. Notably, two samples in the CC group were GynTect^®^ methylation-negative: one was focal adenoid basal cell carcinoma, and the other was carcinoma *in situ* of the cervix. Similarly, CISCER^®^ also demonstrated an upward trend in methylation-positive rates, rising from 3.85% in the CIN1 group to 86.36% in the CC group. Three samples in the CC group were CISCER^®^ methylation-negative, including focal adenoid basal cell carcinoma, carcinoma in situ, and small cell neuroendocrine carcinoma of the cervix. Despite these trends, no significant differences were observed in methylation-positive rates between GynTect^®^ and CISCER^®^ across cervicitis, various grades of cervical lesions, or even cervical cancer. Additionally, we analyzed the test scores of both reagents and the positive rates of each gene in different pathological groups (Supplementary Table 1). ZNF671 and PAX1 demonstrated similar positive rates in GynTect^®^ and CISCER^®^ assay, respectively. DLX1 exhibited the highest positive rate among the six genes tested in GynTect^®^. Regardless of the reagent used, methylation test scores progressively increased with the severity of cervical lesions. Additionally, the median of ΔCt PAX1 and ΔCt JAM3 methylation were significantly different between CIN2 and CIN3 (all *p* < 0.01) as well as between CIN3 and CC group (all *p* < 0.0001) (Supplementary Figure 1).

To investigate whether age influences the methylation-positive rates of the two reagents in different cervical lesions, patients were divided into two age groups: under 30 years old and 30 years old or older, based on the WHO cervical cancer screening guidelines (Table 2). The study revealed that in the under-30 group, from the cervicitis group to the CIN3 group, only CIN3 samples exhibited positive methylation results with both tests, and no significant differences were observed between the two reagents. In the 30 years and older group, from the cervicitis group to the CC group, the methylation positive rate of both reagents demonstrated a gradually increasing trend; however, there was no significant difference in the methylation positive rate of the two reagents within the same cervical lesion category. It is worth noting that among six hrHPV E6/E7 mRNA-negative specimens, which included cervicitis (2 cases), CIN1 (2 cases), CIN2 (1 case), and CIN3 (1 case), one CIN3 specimen tested positive for CISCER^®^ methylation but negative for GynTect^®^ (Figure 1B).

**Table 2:** Methylation positive rates of GynTect^®^ and CISCER^®^ tests in different cervical lesions in women under and over 30 years of age.

Age group	Cervicitis (*n* = 14)	CIN1 (*n* = 26)	CIN2 (*n* = 23)	CIN3 (*n* = 34)	CC (n = 22)	*P* value
Methylation positivity rate, <30 years, % (n/N)						
GynTect^®^	0.00 (0/4)	0.00 (0/7)	0.00 (0/5)	28.57 (2/7)	0 (0/0)	0.1713
CISCER^®^	0.00 (0/4)	0.00 (0/7)	0.00 (0/5)	42.86 (3/7)	0 (0/0)	0.0484
*P* value	1	1	1	1	-	
Methylation positivity rate, ≥30 years, % (n/N)						
GynTect^®^	0.00 (0/10)	10.53 (2/19)	33.33 (6/18)	70.37 (19/27)	90.91(20/22)	<0.0001
CISCER^®^	10.00 (1/10)	5.26 (1/19)	38.89 (7/18)	77.78 (21/27)	86.36 (19/22)	<0.0001
*P* value	1	1	0.7286	0.5346	0.635	

### 3.3 Methylation positive rate in two assays based on cytological grades and HPV status

**Figure 2:**
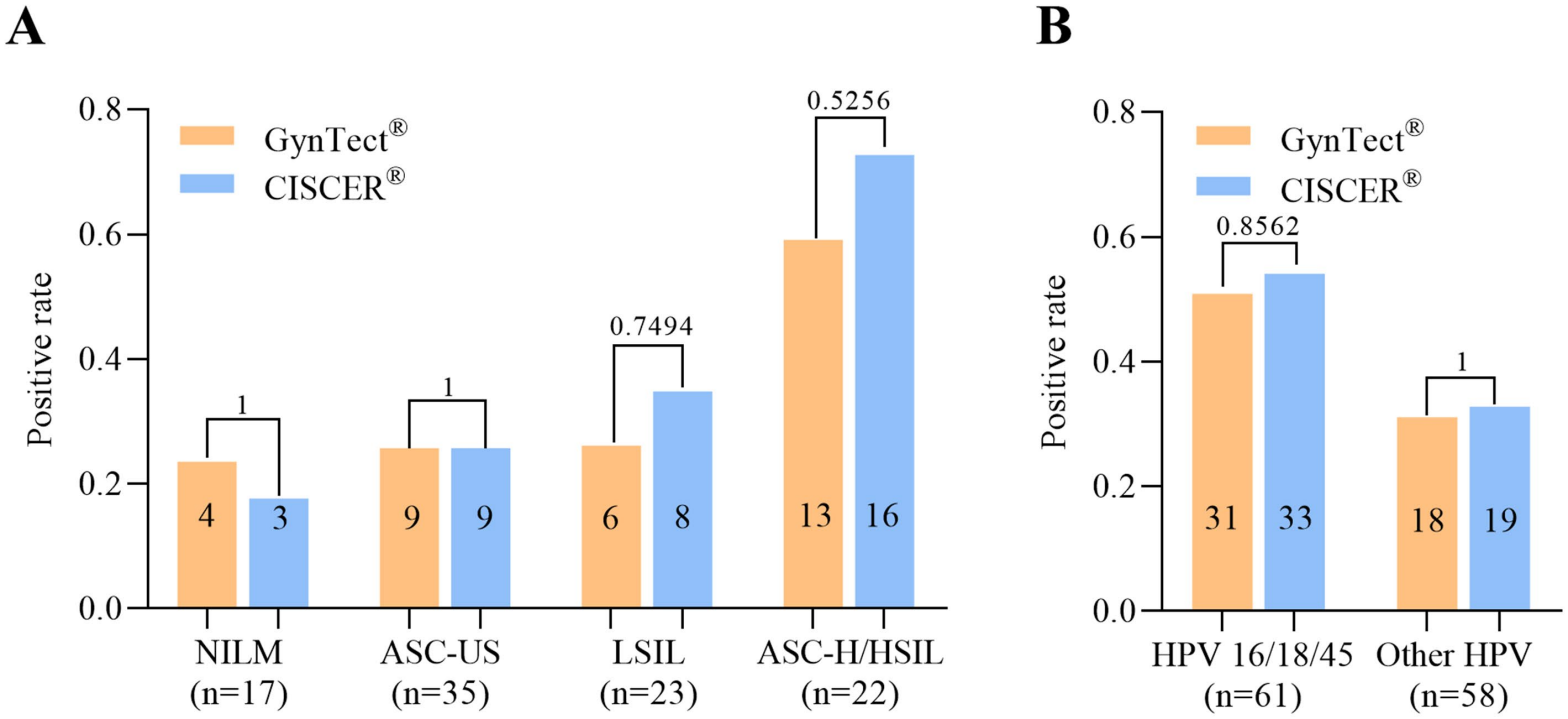
The positive rates of two methylation detection reagents in different cytological and HPV type results. **(A)** The positive rates of GynTect^®^ and CISCER^®^ methylation reagents in NILM, ASC-US, LSIL and ASC-H/HSIL. **(B)** The positive rates of GynTect^®^ and CISCER^®^ methylation reagents in HPV 16/18/45 and other HPV. NILM, Negative for Intraepithelial Lesion or Malignancy; ASC-US, Atypical Squamous Cells of Undetermined Significance; LSIL, Low-grade Squamous Intraepithelial Lesion; ASC-H, Atypical Squamous Cells, cannot exclude HSIL; HSIL, High-grade Squamous Intraepithelial Lesion. The comparisons between two groups were performed with Fisher’s exact test and *p* values less than 0.05 differences were considered statistically significant.

Both GynTect^®^ and CISCER^®^ assays demonstrated varying methylation positive rates across different cervical cytological grades (Figure 2A). Overall, the methylation positivity rate tended to increase with the severity of cervical cytopathology, with this trend being more pronounced in CISCER^®^ (GynTect^®^ *p* = 0.0320 vs. CISCER^®^ *p* = 0.0008). Individually, there were no significant differences in methylation positive rates between the two reagents across the four cytological grades, as all *p* values exceeded 0.05. Specifically, in patients with NILM, GynTect^®^ demonstrated a detection rate of 23.53%, whereas CISCER^®^ showed a slightly lower rate of 17.65% (*p* = 1). For patients with ASC-US, both assays performed similarly, each achieving a detection rate of 25.71%. In the LSIL group, GynTect^®^ identified 26.09% of cases, while CISCER^®^ detected 34.78% (*p* = 0.7494). Among patients with ASC-H/HSIL, GynTect^®^ exhibited a positive detection rate of 59.09%, whereas CISCER^®^ achieved a higher rate of 72.73% (*p* = 0.5256).

When analyzing the performance of assays based on HPV genotype, both GynTect^®^ and CISCER^®^ exhibited higher detection rates for HPV 16/18/45 compared to non-16/18/45 HPV types (GynTect^®^ *p* = 0.0401 vs. CISCER^®^ *p* = 0.0263) (Figure 2B). Specifically, for HPV 16/18/45, GynTect^®^ achieved a detection rate of 50.82%, while CISCER^®^ demonstrated a slightly higher but not statistically significant detection rate of 54.10% (*p* = 0.8562). In contrast, for non-16/18 HPV types, GynTect^®^ detected 31.03% of cases, whereas CISCER^®^ identified 32.76% (*p* = 1). No statistically significant differences were observed between the two assays for either HPV 16/18/45 or non-16/18/45 HPV types.

### 3.4 Clinical performance based on colposcopy results

We evaluated the clinical performance of two commercial methylation-specific PCR assays (GynTect^®^ and CISCER^®^), for triaging hrHPV E6/E7 mRNA positive women across various pathological stages, including cervicitis, CIN1, CIN2, CIN3 and CC (Table 3). For CIN2 + lesions, GynTect^®^ demonstrated a positive rate of 59.49%, while CISCER^®^ showed a rate of 63.29%. Both assays exhibited a specificity of 95.00%. For CIN3 + lesions, GynTect^®^ demonstrated a sensitivity of 73.21%, specificity of 87.30%, a positive predictive value (PPV) of 83.7%, and a negative predictive value (NPV) of 78.6%, whereas CISCER^®^ showed a sensitivity of 76.79%, specificity of 85.71%, a PPV of 82.7%, and an NPV of 80.6%. Statistical analysis indicated no significant differences in sensitivity between the two assays for detecting CIN2 + (*p* = 0.6240) or CIN3 + (*p* = 0.6625) lesions. Similarly, no statistically significant differences were observed in specificity for CIN2 + (*p* = 1) or CIN3 + (*p* = 0.7943) lesions. These results suggest that GynTect^®^ and CISCER^®^ offer comparable clinical performance in identifying high-grade cervical intraepithelial neoplasia among hrHPV E6/E7 mRNA positive women, although CISCER^®^ exhibited slightly higher sensitivity in detecting both CIN2 + and CIN3 + lesions.

**Table 3:** Clinical performance of both assays regarding the detection of CIN2 + and CIN3 + in the hrHPV E6/E7 mRNA positive patients.

Assay	True positive	Sensitivity [%]	True negative	Specificity [%]	PPV [%]	NPV [%]
CIN2+						
GynTect^®^	47/79	59.49	38/40	95.00	95.9	54.3
CISCER^®^	50/79	63.29	38/40	95.00	96.2	56.7
*P* value		0.624		1	0.952	0.775
CIN3+						
GynTect^®^	41/56	73.21	55/63	87.30	83.7	78.6
CISCER^®^	43/56	76.79	54/63	85.71	82.7	80.6
*P* value		0.662		0.794	0.895	0.769

PPV, positive predictive value; NPV, negative predictive value.

Furthermore, the sensitivity and specificity of all methylated genes included in the two assays were separately evaluated for CIN2 + and CIN3 + (Supplementary Table 2). Among the 6 gene GynTect^®^ panel, DLX1 exhibited the highest sensitivity and lowest specificity in both CIN2 + and CIN3+, whereas SOX17 demonstrated the lowest sensitivity and highest specificity. ZNF671 showed performance comparable to the overall GynTect^®^ reagents in terms of sensitivity and specificity. In the 2 gene CISCER^®^ panel, PAX1 displayed sensitivity and specificity levels similar to those of the CISCER^®^ reagents, while JAM3 had relatively lower sensitivity and higher specificity for both CIN2 + and CIN3 + .

The odds ratio (OR) of the two assays was analyzed in CIN2 + and CIN3 + compared with CIN1 (Table 4). The ORs of GynTect^®^ and CISCER^®^ reagents in CIN2 + were 17.63 (95% CI 3.890–79.85) and 43.10 (95% CI 5.546–335.0), respectively. The ORs of GynTect^®^ and CISCER^®^ reagents in CIN3 + were 32.80 (95% CI 6.899–155.9) and 82.69 (95% CI 10.20–670.5), respectively. Among the six genes in the GynTect^®^ reagent, ZNF671 exhibited the highest OR in both CIN2 + (OR 16.73, 95% CI 3.694–75.74) and CIN3 + (OR 32.80, 95% CI 6.899–155.9). Among the two genes in the CISCER^®^ reagent, PAX1 demonstrated the highest OR in both CIN2 + (OR 40.83, 95% CI 5.258–317.1) and CIN3 + (OR 75.00, 95% CI 9.292–605.3).

**Table 4:** The risks of CIN2 + and CIN3 + in GynTect^®^ and CISCER^®^.

Methylation marker	CIN1 (*n* = 40)	CIN2 + (*n* = 79)	CIN3 + (*n* = 56)
GynTect^®^ OR (95% CI)	Reference	17.63 (3.890–79.85)	32.80 (6.899–155.9)
*P* value	-	<0.0001	<0.0001
ASTN1 OR (95% CI)	Reference	3.419 (1.241–9.418)	5.556 (1.923–16.05)
*P* value	-	0.014	0.0009
DLX1 OR (95% CI)	Reference	4.098 (1.582–10.62)	6.750 (2.411–18.89)
*P* value	-	0.0026	0.0001
ITGA4 OR (95% CI)	Reference	6.960 (1.532–31.61)	9.677 (2.084–44.95)
*P* value	-	0.0049	0.0009
RXFP3 OR (95% CI)	Reference	1.731 (0.622–4.819)	2.323 (0.808–6.681)
*P* value	-	0.2903	0.1127
SOX17 OR (95% CI)	Reference	12.26 (1.574–95.57)	18.75 (2.372–148.2)
*P* value	-	0.0033	0.0004
ZNF671 OR (95% CI)	Reference	16.73 (3.694–75.74)	32.80 (6.899–155.9)
*P* value	-	<0.0001	<0.0001
CISCER^®^ OR (95% CI)	Reference	43.10 (5.546–335.0)	82.69 (10.20–670.5)
*P* value	-	<0.0001	<0.0001
PAX1 OR (95% CI)	Reference	40.83 (5.258–317.1)	75.00 (9.292–605.3)
*P* value	-	<0.0001	<0.0001
JAM3 OR (95% CI)	Reference	29.86 (3.855–231.3)	62.50 (7.799–500.9)
*P* value	-	<0.0001	<0.0001

OR, odds ratio.

### 3.5 The correlation between the positive result of the two assays and prognosis

**Figure 3:**
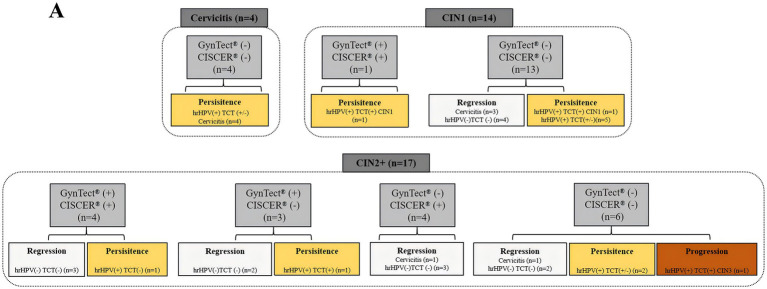
The correlation between the results of two methylation-based cervical cancer detection assays. TCT, Thinprep Cytologic Test.

The correlation between the results of two methylation-based cervical cancer detection assays, GynTect^®^ and CISCER^®^, and patient prognosis was evaluated in 35 patients who underwent cervical conserving treatments over a period of one to 2 years (Figure 3). These treatments included observation, medication guidance, laser therapy, loop electrosurgical excision procedure (LEEP), and conization. Among the 35 patients, four patients diagnosed with cervical inflammation exhibited negative results for both both GynTect^®^ and CISCER^®^. Follow-up data indicated that these four patients remained diagnosed with cervicitis and demonstrated persistent hrHPV positivity. Furthermore, among the 14 patients with CIN1, one patient tested positive with both assays and was still diagnosed with CIN1, showing hrHPV positivity and abnormal cytology during follow-up. The remaining 13 CIN1 patients were negative for both agents, including 3 cases of pathological regression, 4 cases of hrHPV and cytological regression, 1 case of persistent CIN1 with hrHPV positivity and abnormal cytology, and 5 cases of persistent hrHPV-positive cervix with or without normal cytology. Among the remaining 17 patients diagnosed with CIN II or CIN III, 4 patients with positive GynTect^®^ and CISCER^®^ results included 1 case of persistent hrHPV positivity, while the other 3 patients showed regression with negative hrHPV and normal cytology. Of the 3 patients with positive GynTect^®^ results, 2 cases showed regression with negative hrHPV and normal cytology, and 1 patient exhibited persistent cytological abnormalities and hrHPV positivity. Among the 4 patients with positive CISCER^®^ results, all cases showed regression. Among the 6 patients with negative GynTect^®^ and CISCER^®^ results, 3 cases showed regression, 2 cases remained hrHPV positive with or without normal cytology, and 1 case progressed to CIN3 with hrHPV positivity and abnormal cytology. Due to the limited sample size of the follow-up study, it remains uncertain whether there is a true association between the methylation results of these two assays and patient prognosis.

## 4 Discussion

As cervical cancer screening shifts from cytology to HPV-based methods, low specificity of hrHPV testing has increased colposcopy referrals and overtreatment. Accurate triage methods are urgently needed to distinguish clinically relevant lesions from transient infections (22–24). In this study, we compared the performance of the GynTect^®^ and CisCer^®^ methylation assays in hrHPV E6/E7 mRNA–positive specimens. Both assays demonstrated strong performance in detecting high-grade cervical lesions, with comparable effectiveness in risk stratification. Notably, Ciscer showed advantages in terms of cost-effectiveness and operational simplicity, supporting its suitability for broader clinical application.

To our knowledge, limited data exist on DNA methylation assays in hrHPV E6/E7 mRNA–positive women, and our findings contribute to this underexplored area. Unlike HPV DNA testing, which only indicates viral presence, E6/E7 mRNA detection reflects active viral oncogene expression and therefore correlates more closely with lesion severity and cancer progression risk. Previous studies demonstrated that E6/E7 mRNA testing achieves a sensitivity comparable to HPV DNA testing, while offering greater specificity for detecting HSIL (25, 26). Women positive for E6/E7 mRNA are therefore considered at higher risk and may benefit from more targeted triage approaches (16). In this study, both DNA methylation–based assays demonstrated improved diagnostic performance in hrHPV E6/E7 mRNA–positive women, with sensitivities of 73.21% (GynTect^®^) and 76.79% (CisCer^®^), specificities of ~85–87%, PPV of ~82.7–83.7%, and NPV of ~78.6–80.6%. These findings highlighted the potential clinical value of integrating methylation triage with mRNA-based screening for more precise and efficient risk stratification.

The baseline HPV detection methods, study settings, and triaging populations contribute to the reported variations in triage performance (12, 27, 28). Our sensitivities (~73–77%) and specificities (~85–87%) are broadly similar to those reported by Shang et al. in HPV DNA–positive women with minimally abnormal cytology (sensitivity 74.4% and specificity 88.8%) (19), indicating that the assays maintain stable accuracy in detecting disease and excluding non-disease, and highlighting their robustness across different risk-selected contexts. Furthermore, our cohort was restricted to hrHPV E6/E7 mRNA–positive women, who represent a biologically higher-risk group with enriched CIN3 + prevalence and stronger methylation signals. This pre-selection increases the practical value of a positive test result, as reflected by a higher PPV (82.7% vs. 40.1%). Clinically, this enrichment effect implies that methylation triage in mRNA-positive women may offer greater efficiency by reducing unnecessary colposcopy referrals.

On the other hand, our findings on GynTect^®^ and CISCER^®^ are consistent with broader evidence from other DNA methylation assays, such as the S5 classifier, which quantifies methylation in the host gene *EPB41L3* as well as viral regions of HPV16, HPV18, HPV31, and HPV33, demonstrating high sensitivity (89.1%) and specificity (76.6%) for “≥HSIL+” in triaging women with abnormal HPV/cytology results (29). Unlike GynTect^®^ and CISCER^®^, which focus on specific host genes, the S5 assay incorporates both host and viral markers, potentially enhancing risk stratification for CIN3 + lesions. Future comparative studies are warranted to determine the most effective assay for clinical implementation, taking into account factors such as cost, operational simplicity, and diagnostic performance.

Our study demonstrated a progressive increase in methylation positivity rates from cervicitis to cervical cancer—rising from 0 to 90.91% with GynTect^®^ and from 3.85 to 86.36% with CisCer^®^. This trend underscores the dynamic role of DNA hypermethylation in HPV-driven cervical carcinogenesis and aligns with previous findings (27). While most studies report nearly 100% methylation positivity in cervical cancer, three of our 22 cancer cases were negative. These included one adenoid basal cell carcinoma and one carcinoma *in situ* (both negative for GynTect^®^ and CisCer^®^), and one small cell neuroendocrine carcinoma (negative for CisCer^®^ only). In the adenoid basal cell carcinoma case, the initial biopsy suggested CIN3, and cancer was only confirmed after surgical excision. The negative result may be due to a low tumor cell proportion, with methylation signals diluted by normal or inflammatory cells. Importantly, both assays incorporate methylation markers that are particularly sensitive to squamous cell carcinoma, which may limit their detection capacity in other histological subtypes. Although carcinoma in situ is a high-grade lesion, its methylation levels may fall below assay thresholds due to incomplete methylation and focal distribution. For instance, PAX1 methylation is often elevated in invasive cancers but only partially detected in CIS. Moreover, small cell neuroendocrine carcinoma exhibits a distinct methylation profile and previous studies have reported negative PAX1 methylation in these tumors (30). These findings underscore the importance of histology-specific validation when applying methylation markers in cervical cancer screening and triage.

Higher methylation positivity rates in women aged ≥30 years, especially for advanced lesions, strongly support WHO’s recommendation for age-stratified cervical cancer screening. In younger women (<30 years), methylation positivity was observed only in CIN3 cases, likely reflecting transient HPV infections and the higher spontaneous regression rates of low-grade lesions (31). In contrast, persistent hrHPV infection in older women leads to cumulative epigenetic changes, resulting in increased methylation detection.

Both assays showed higher detection rates for HPV16/18/45-associated lesions (GynTect^®^: 50.82%; CisCer^®^: 54.10%), reflecting the stronger oncogenicity of these genotypes (32). However, their relatively low sensitivity for non-16/18/45 types (31–33%) highlights a limitation, especially as genotypes like HPV52 and 58 are increasingly linked to cervical cancer in certain populations (5). Adding genotype-specific methylation markers to the assays could improve their overall diagnostic performance.

While both assays demonstrated comparable sensitivity for detecting CIN3 + lesions (GynTect^®^: 73.21%, CisCer^®^: 76.79%) and specificity (~85–87%), CisCer^®^ exhibited slightly higher odds ratios for CIN2 + (OR 43.10 vs. 17.63) and CIN3 + (OR 82.69 vs. 32.80), suggesting stronger predictive value for advanced lesions. Although GynTect^®^ has been reported to detect as little as 0.1 ng of genomic DNA (27), 8 samples in our study yielded invalid results and were excluded from analysis. In addition, the GynTect^®^ panel involves six methylation targets and two internal controls processed in separate wells, increasing both testing cost and technical burden. In contrast, CisCer^®^ only showed 2 invalid results and employs a multiplex real-time PCR approach targeting only two genes (PAX1 and JAM3), enabling a simpler, more efficient workflow. Given its comparable diagnostic performance and operational advantages, CisCer^®^ may be better suited for broader clinical implementation. Refining gene panels and marker combinations may further improve diagnostic performance and clinical utility. In addition, a key advantage of methylation testing is its ability to use the same sample as the initial HPV test, and self-collected samples have shown great potential for expanding screening access. Future studies should further explore the use of self-sampling to enable efficient one-visit screening and triage, particularly in resource-limited settings.

The superior performance of specific genes such as PAX1 (CisCer^®^) and ZNF671 (GynTect^®^) highlights their utility as standalone markers, whereas the relatively lower specificity of DLX1 (GynTect^®^) may be attributed to its broader methylation patterns observed in both precancerous and inflammatory tissues (33, 34). PAX1 methylation has been reported to outperform HPV16/18 genotyping and achieve comparable clinical performance to cytology (35). Additional methylation markers are currently under investigation. For instance, ZNF582 methylation has demonstrated superior performance compared to PAX1 in detecting CIN3 + among hrHPV-infected women (36). Furthermore, marker performance varies by histology: PAX1 is more sensitive for squamous cell carcinoma, while JAM3 better detects adenocarcinoma and rare cervical tumors. Combining these markers may improve coverage across cervical cancer subtypes.

Given the limited sample size of the follow-up cohort, the relationship between methylation results of the two assays and patient prognosis could not be conclusively determined. (e.g., regression or persistence). Notably, one CIN3 patient with a negative methylation result progressed to persistent disease, indicating that a negative result does not entirely exclude residual risk. The WHO recommends retesting triage-negative women (excluding WLHIV) after 24 months (37). Previous studies reported a 3.5% cumulative incidence of CIN3 + over a 3-year follow-up among hrHPV-positive women, which decreased to 2.1% in those with a negative GynTect methylation result and further to 1.6% when combined with HPV16/18 genotyping (38). These findings highlight the long-term protective value of methylation-based triage (39). Further large-scale prospective studies are warranted to evaluate the prognostic utility of methylation testing, particularly in conservatively managed patients.

Our study identified an intriguing case of histologically confirmed CIN3 that tested negative for hrHPV E6/E7 mRNA yet was positive by the CISCER^®^ methylation assay. This discordance may be explained by several biologically plausible mechanisms: (1) a ‘molecular scar’ from a prior cleared HPV infection, in which viral oncogene-driven methylation changes persist despite viral clearance; (2) infection with a rare or novel HPV genotype not targeted by the mRNA assay; or (3) a very low viral load or mRNA expression level below the assay’s detection threshold in a true high-grade lesion. Although the possibility of a false-positive methylation result cannot be entirely ruled out, the positivity in this specimen was limited to *JAM3*. In our study, *JAM3* hypermethylation showed high specificity for high-grade CIN, making this explanation less likely. This finding highlights the potential of DNA methylation markers to serve as persistent indicators of oncogenic transformation, potentially identifying high-risk lesions that might be missed when relying solely on HPV detection.

Our clinical sample collection was not derived from a primary screening population; therefore, the diagnostic performance reported here may not directly apply to primary screening settings. Future studies should focus on validating these assays in diverse populations with varying prevalence of non-16/18 HPV genotypes; investigating longitudinal changes in methylation patterns to predict lesion progression or regression; and further establishing consistent data on the cost-effectiveness of DNA methylation testing, especially in resource-limited settings. The sample size, particularly when stratified into pathological subgroups, represents a limitation that affects the precision of our estimates. Future validation in a larger, multi-center prospective study is warranted to confirm these results and enhance their generalizability. In addition, the emergence of novel cervical cancer markers, such as the vaginal microbiome (40) and host immune responses to HPV infection (41), may influence disease progression and potentially affect the performance of methylation-based triage strategies. Integrating these variables into future studies could help refine risk stratification and improve the overall effectiveness of cervical cancer screening programs.

## 5 Conclusion

GynTect^®^ and CisCer^®^ exhibit robust performance in identifying high-grade cervical lesions in hrHPV E6/E7 mRNA–positive women, with methylation levels serving as reliable indicators of disease severity and HPV oncogenic potential. Although neither assay is designed to predict short-term prognosis, their high specificity underscores their value in minimizing overtreatment. Further refinement of marker panels and integration of multimodal strategies will enhance their utility in precision cervical cancer screening.

## Data Availability

The original contributions presented in the study are included in the article/Supplementary material, further inquiries can be directed to the corresponding author.
