# Supplementary material for: Comparative evaluation of two DNA methylation assays for triage of hrHPV E6/E7 mRNA–positive women
Source: Front Public Health. 2025 Nov 21;13:1723553. doi: 10.3389/fpubh.2025.1723553 (PMC12678232; doi:10.3389/fpubh.2025.1723553)
Supplement: Supplementary file 1 [file Table_1.docx]

**Supplementary Table 1.** The positive rate of every methylation marker in cervical precancerous and cancerous lesions.

.

| **Methylation marker, % (n)** | **Cervicitis (n=14)** | **CIN1**  **(n=26)** | **CIN2**  **(n=23)** | **CIN3**  **(n=34)** | **CC**  **(n=22)** | ***P* value** |
| --- | --- | --- | --- | --- | --- | --- |
| **GynTect^®^** | **0.00 (0)** | **7.69 (2)** | **26.09 (6)** | **61.76 (21)** | **90.91 (20)** | <0.001 |
| ASTN1 | 14.29 (2) | 23.08 (6) | 21.74 (5) | 44.12 (15) | 90.91 (20) | <0.001 |
| DLX1 | 71.43 (10) | 30.77 (8) | 39.13 (9) | 61.76 (21) | 95.45 (21) | 0.002 |
| ITGA4 | 0.00 (0) | 7.69 (2) | 17.39 (4) | 32.35 (11) | 63.64 (14) | <0.001 |
| RXFP3 | 0.00 (0) | 23.08 (6) | 17.39 (4) | 17.65 (6) | 77.27 (17) | <0.001 |
| SOX17 | 0.00 (0) | 3.85 (1) | 8.70 (2) | 23.53 (8) | 72.73 (16) | <0.001 |
| ZNF671 | 0.00 (0) | 7.69 (2) | 21.74 (5) | 61.76 (21) | 90.91 (20) | <0.001 |
| Scores (Mean±SD) | 0.86±0.66 | 1.12±1.73 | 1.70±2.14 | 3.71±2.83 | 6.73±1.91 | <0.0001 |
| **CISCER^®^** | **7.14 (1)** | **3.85 (1)** | **30.43 (7)** | **70.59 (24)** | **86.36 (19)** | <0.001 |
| PAX1 | 7.14 (1) | 3.85 (1) | 30.43 (7) | 67.65 (23) | 86.36 (19) | <0.001 |
| JAM3 | 0.00 (0) | 3.85 (1) | 13.04 (3) | 61.76 (21) | 86.36 (19) | <0.001 |
| Scores (Mean±SD) | 0.07±0.27 | 0.08±0.39 | 0.43±0.73 | 1.29±0.91 | 1.73±0.70 | <0.0001 |
