# Supplementary material for: Comparative evaluation of two DNA methylation assays for triage of hrHPV E6/E7 mRNA–positive women
Source: Front Public Health. 2025 Nov 21;13:1723553. doi: 10.3389/fpubh.2025.1723553 (PMC12678232; doi:10.3389/fpubh.2025.1723553)
Supplement: Supplementary file 2 [file Table_2.docx]

| **Methylation marker** | **True positive** | **Sensitivity % (95%CI)** | **True negative** | **Specificity % (95%CI)** |
| --- | --- | --- | --- | --- |
| **CIN2+** |  |  |  |  |
| GynTect^®^ | 47/79 | 59.49 (47.86-70.40) | 38/40 | 95.00 (83.08-99.39) |
| ASTN1 | 40/79 | 50.63 (39.14-62.08) | 32/40 | 80.00 (64.35-90.95) |
| DLX1 | 51/79 | 64.56 (52.99-75.00) | 22/40 | 55.00 (38.49-70.74) |
| ITGA4 | 29/79 | 36.71 (26.14-48.31) | 38/40 | 95.00 (83.08-99.39) |
| RXFP3 | 27/79 | 34.18 (23.87-45.71) | 34/40 | 85.00 (70.16-94.29) |
| SOX17 | 26/79 | 32.90 (22.70-44.40) | 39/40 | 97.50 (86.80-99.90) |
| ZNF671 | 46/79 | 58.20 (46.60-69.20) | 38/40 | 95.00 (83.08-99.39) |
| CISCER^®^ | 50/79 | 63.29 (51.69-73.86) | 38/40 | 95.00 (83.08-99.39) |
| PAX1 | 49/79 | 62.00 (50.40-72.70) | 38/40 | 95.00 (83.08-99.39) |
| JAM3 | 43/79 | 54.40 (42.80-65.70) | 39/40 | 97.50 (86.80-99.90) |
| **CIN3+** |  |  |  |  |
| GynTect^®^ | 41/56 | 73.21 (59.70-84.17) | 55/63 | 87.30 (76.50-94.35) |
| ASTN1 | 35/56 | 62.50 (48.55-75.08) | 50/63 | 79.37 (67.30-88.53) |
| DLX1 | 42/56 | 75.00 (61.63-85.61) | 36/63 | 57.14 (44.05-69.54) |
| ITGA4 | 25/56 | 44.64 (31.34-58.53) | 57/63 | 90.48 (80.41-96.42) |
| RXFP3 | 23/56 | 41.07 (28.10-55.02) | 53/63 | 84.13 (72.74-92.12) |
| SOX17 | 24/56 | 42.90 (29.70-56.80) | 60/63 | 95.20 (84.70-99.00) |
| ZNF671 | 41/56 | 73.20 (59.70-84.20) | 56/63 | 88.90 (78.40-95.40) |
| CISCER^®^ | 43/56 | 76.79 (63.58-87.02) | 54/63 | 85.71 (74.61-93.25) |
| PAX1 | 42/56 | 75.00 (61.60-85.60) | 54/63 | 85.71 (74.61-93.25) |
| JAM3 | 40/56 | 71.40 (57.80-82.70) | 59/63 | 93.70 (84.50-98.20) |

**Supplementary Table 2.** Clinical performance of all methylated genes included in GynTect^®^ and CISCER^®^ assays regarding the detection of CIN2+ and CIN3+ in the HPV E6/E7 mRNA positive patients.(n = 119).
