# Supplementary figures and images for: Comparative evaluation of two DNA methylation assays for triage of hrHPV E6/E7 mRNA–positive women
Source: Front Public Health. 2025 Nov 21;13:1723553. doi: 10.3389/fpubh.2025.1723553 (PMC12678232; doi:10.3389/fpubh.2025.1723553)

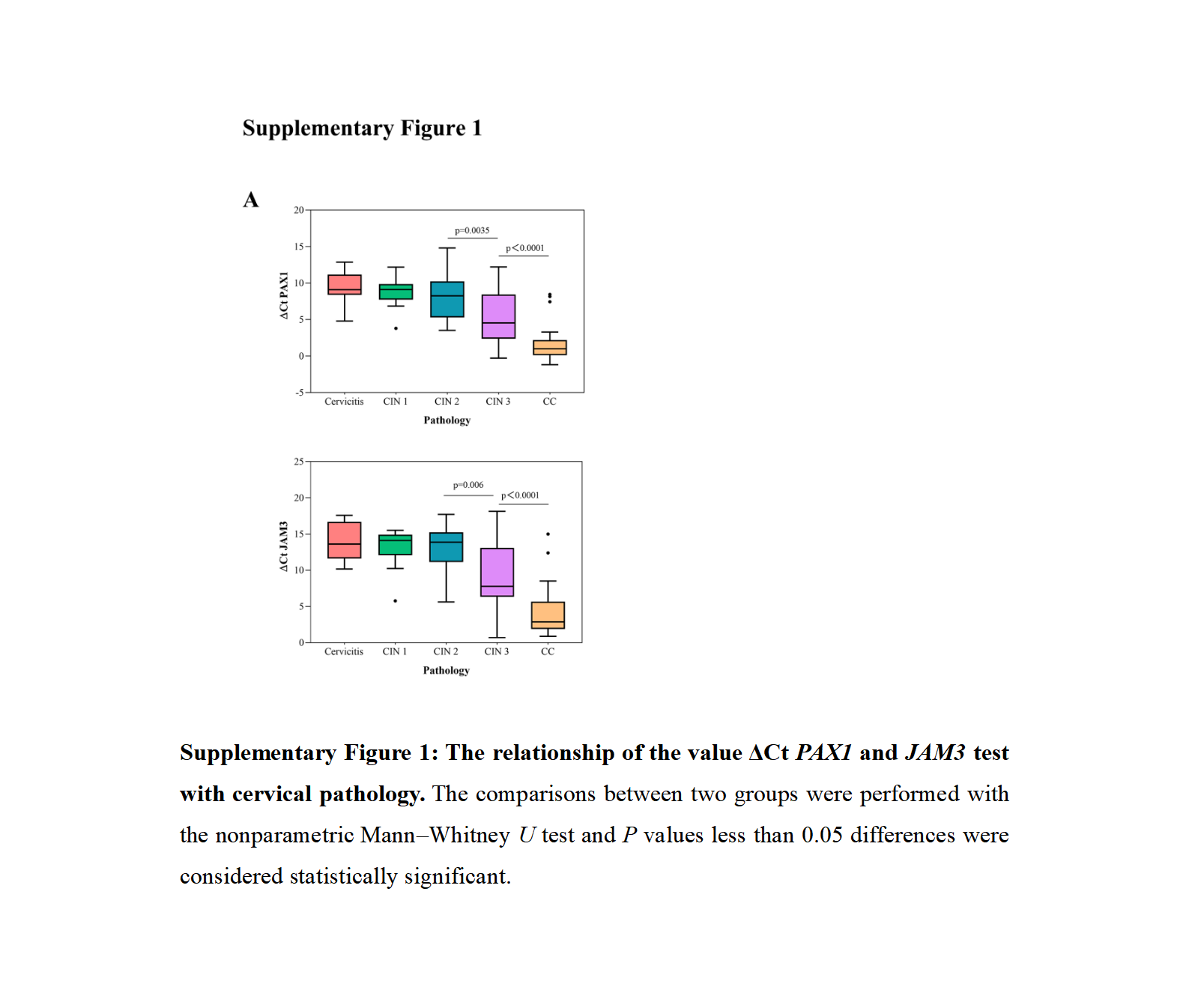

Supplement: Supplementary file 3 [file Image_1.tif]
